# Autonomous Vehicles: Disengagements, Accidents and Reaction Times

**DOI:** 10.1371/journal.pone.0168054

**Published:** 2016-12-20

**Authors:** Vinayak V. Dixit, Sai Chand, Divya J. Nair

**Affiliations:** Research Centre for Integrated Transport Innovation (rCITI), School of Civil and Environmental Engineering, UNSW Australia, Sydney New South Wales, Australia; Beihang University, CHINA

## Abstract

Autonomous vehicles are being viewed with scepticism in their ability to improve safety and the driving experience. A critical issue with automated driving at this stage of its development is that it is not yet reliable and safe. When automated driving fails, or is limited, the autonomous mode disengages and the drivers are expected to resume manual driving. For this transition to occur safely, it is imperative that drivers react in an appropriate and timely manner. Recent data released from the California trials provide compelling insights into the current factors influencing disengagements of autonomous mode. Here we show that the number of accidents observed has a significantly high correlation with the autonomous miles travelled. The reaction times to take control of the vehicle in the event of a disengagement was found to have a stable distribution across different companies at 0.83 seconds on average. However, there were differences observed in reaction times based on the type of disengagements, type of roadway and autonomous miles travelled. Lack of trust caused by the exposure to automated disengagements was found to increase the likelihood to take control of the vehicle manually. Further, with increased vehicle miles travelled the reaction times were found to increase, which suggests an increased level of trust with more vehicle miles travelled. We believe that this research would provide insurers, planners, traffic management officials and engineers fundamental insights into trust and reaction times that would help them design and engineer their systems.

## Introduction

Autonomous vehicles are predicted to be transformative, with a potential to improve productivity, reduce congestion and improve safety. However, there are many safety and risk related unknowns associated with the autonomous vehicles, with regards to factors affecting disengagements and driver behaviour, at moments requiring manual resumption of vehicle control. The ultimate success of automated vehicles will depend on drivers’ trust in them and on how people choose to use and interact with them, and the ensuing safety risk.

To date, research has focused predominantly on automation technology [1], acceptance in adopting automated vehicle technology [2,3], the impact of automated vehicles on safety and congestion [4,5] and legal, regulatory and other barriers to implementation [6]. Manufacturers have undertaken human factors research into automated driving [7], but only limited published research addresses driver interactions with automated vehicles [8].

A key issue with automated driving at this stage of its development is that it is not yet reliable and safe [9]. When automated driving fails, or is limited (e.g., the inability of on-board computer algorithms to make a safe decision), the autonomous mode disengages and the drivers are expected to resume manual driving. For this transition to occur safely, it is imperative that drivers react in an appropriate and timely manner [8].

This paper evaluates the trends in safety risks in the emerging automated vehicle technology by exploring factors influencing: (i) disengagements, (ii) accidents, and (iii) driver reaction times, and emergent phenomena. This study utilizes actual field data from trials that are being conducted on Californian public roads, which include freeways, highways and urban streets. It is anticipated that this research would provide engineers, designers and planners an insight into safety aspects of automated driving.

## Data Description

The California Department of Motor Vehicles (DMV) is the state agency that registers motor vehicles, issues permit and monitors the testing of autonomous vehicles. As part of the regulation, companies that are given permits to operate autonomous vehicles are required to file an annual report on disengagements and accidents [10]. As of January 3, 2016, the following companies reported on autonomous vehicle trials from September 2014 to November 2015 in California:

(i) Bosch (ii) Delphi (iii) Google (iv) Mercedes-Benz (v) Nissan (vi) Volkswagen Group (vii) Tesla*

(*Tesla motors submitted a report that claimed that they did not have any disengagements, and did not provide any additional data).

This study used data collated from the disengagement reports of these companies. These disengagement reports contain aggregated information about monthly miles travelled in autonomous mode and number of disengagements. The disengagement reports also provide details on each disengagement, such as road type, weather condition and factors contributing to the disengagements. In addition, Google and Mercedes-Benz reported the accurate reaction times required by the drivers to take control of the vehicle. The other companies that did report reaction times provided upper-bounds (eg. stated as < 1 sec), which was not useful for analysing reaction times.

The disengagements and test drivers have a crucial role in refining the AV technology and ensuring the safe operation of the vehicles since AVs are still in the development phase. The objective of the companies, in general is not exactly minimizing the disengagements, but to gather as much data as possible, while operating safely. The companies intentionally recruit non-software people as test drivers, so as to analyze their experience just like an average driver would, not through the lens of an engineer. They undergo a rigorous formal training process (as reported by Google to California DMV) where they prove their skills in normal and challenging conditions such as a rain simulator, a traffic circle, and other test hazards. They are constantly alert and are directed to take control of the vehicle as often as they feel necessary and for a variety of reasons relating to the comfort of the ride, the safety of the vehicle, or the erratic or unpredictable behaviour of other road users.

As part of the DMV regulations, the companies are also required to provide a report of a traffic accident involving an autonomous vehicle within ten business days of the incident. The DMV website provides access to the police reports of the accidents from September 2014 to as recently as February 2016. Except for the one latest accident, in all these accidents the autonomous vehicle was not at fault. During the annual reporting period from September 2014-November 2015, all accidents involved the autonomous vehicles being not-at-fault. Two other accidents happened after the reporting period in January and February of 2016.

## Analysis

**Fig 1:**
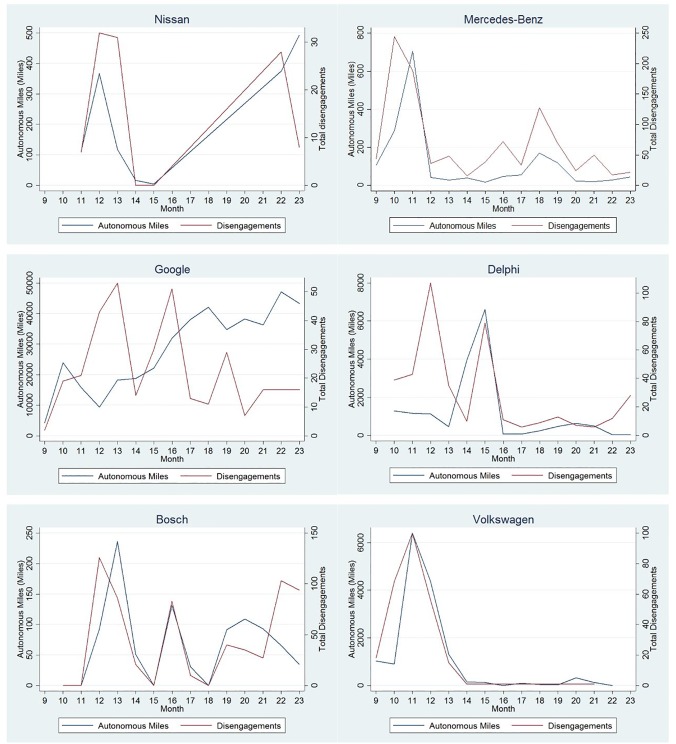
Monthly autonomous miles travelled by different companies.

There was a wide variation in autonomous miles travelled among the companies in California during the considered period, which is shown in Fig 1. Based on the data provided by the companies, a total of 460,097 autonomous miles were driven during the testing period from September 2014 to November 2015. Google accumulated a total of 424,331 autonomous miles which accounts to 92% of total autonomous miles travelled by all reported autonomous driving on California roads.

We use the *autonomous miles* travelled as a measure of exposure for disengagements and accidents in autonomous mode. It should be expected that the exposure of the vehicle to risks will be significantly different when it is being driven by the automation software and the driver. In this research, we calculate disengagement exposure as well as accident exposure per autonomous miles. In the future, safety analysis and modelling should require measuring the exposure in two domains, i.e. *autonomous miles* travelled as a function of software and the *driver miles* travelled.

### Disengagements

Disengagements pose a risk in automated vehicles requiring drivers to be alert and capable of taking control of the vehicle. On an average, the number of disengagement exposure ranged from approximately 1.1 disengagements per 1000 miles for Google to 980 disengagements per 1000 miles for Mercedes-Benz. Towards the end of this reporting period, Google had consistently the lowest disengagement exposure at around 0.18 disengagements per 1000 miles. The total number of monthly disengagements and monthly autonomous miles for each company is shown in Fig 1.

The disengagements can be broadly classified as automatic and manual disengagements. Automatic disengagements are disengagements resulting from the system recognizing a failure or a potential failure in the ability to ensure safety under automated driving conditions. These failures generally occurred due to failure in detection technology, communications breakdown, improper sensor readings, map or calibration issues, errors in data reception, or even due to some hardware issues. In such a situation, the autonomous vehicle informs the driver of such a failure, in which case the driver needs to take control of the vehicle immediately. On the other hand, manual disengagements are the ones where the human driver takes control of the car on his/her own will. Manual disengagements generally occur when the drivers suspect a precarious situation in response to other road users, due to discomfort with the autonomous mode, adverse weather conditions, construction activities, to perform lane changing in heavy traffic, poor road infrastructure, etc.

#### Relationship between manual and automatic disengagements

**Fig 2:**
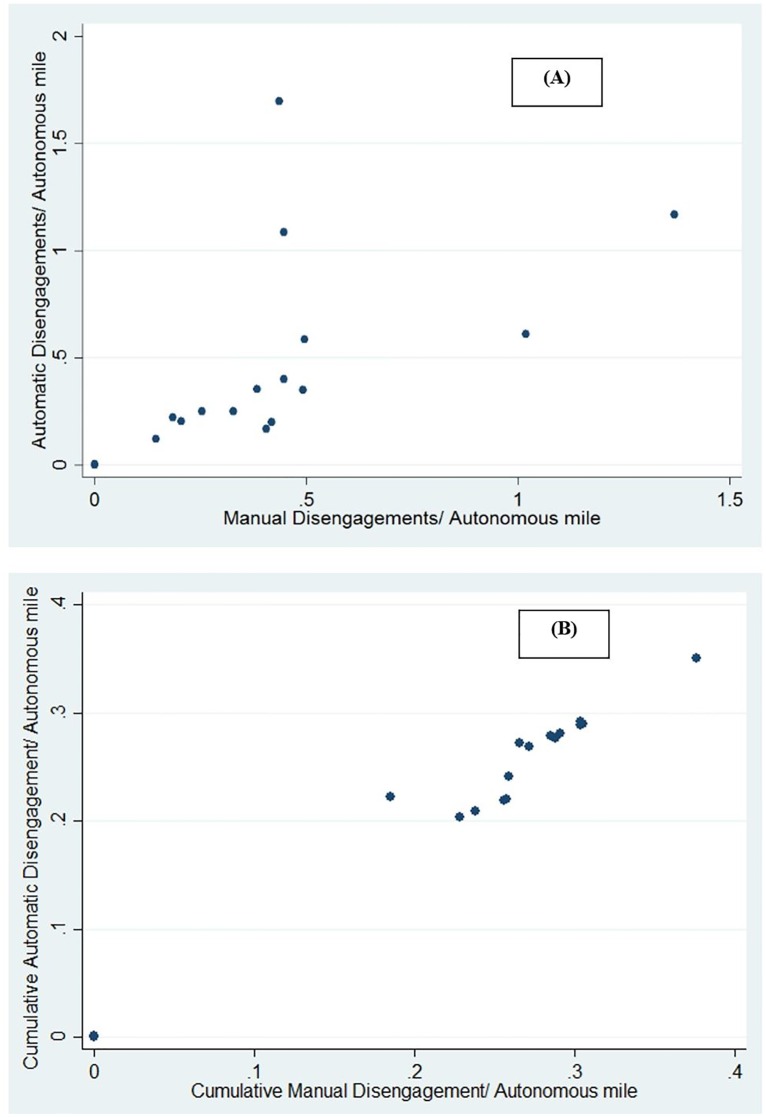
Relationship between exposure of automated and manual disengagements. (A) Monthly automatic disengagements/autonomous miles vs Monthly manual disengagements/autonomous miles. (B) Cumulative automatic disengagements/autonomous miles vs Cumulative manual disengagements/autonomous miles.

In some sense, the number of manual disengagements is a reflection of trust in the automated system to navigate through safely through the driver’s perceived risks. If drivers’ form that trust based on their experience of automatic disengagements, then people should be expected to more likely to manually disengage as the number of automatic disengagements increases. This is confirmed by the high correlation of 0.73 (p-value < 0.01) (Fig 2A) observed between the monthly automatic disengagements/mile and manual disengagements/mile. It should be noted that only Google and Mercedes-Benz reported on the automatic and manual disengagements.

Further, since a drivers’ experience of disengagements are based on cumulative numbers, we also studied the correlation between the cumulative exposure of automated disengagements and manual disengagements. This correlation was found to be significantly higher at 0.82 (p-value < 0.01) (Fig 2B). Cumulative automated (manual) disengagement exposure is calculated as the ratio between the cumulative number of automated (manual) disengagements experienced till each month and the cumulative vehicle miles travelled till each month. Since the manual disengagements occur due to the driver taking control of the vehicle on their own accord, the correlation with the automated disengagements can be mainly attributed to trust.

Google carried out further analysis to study the underlying risks that resulted in driver initiated disengagements. They reported manual disengagements only when the safe operation of the vehicle requires that the autonomous vehicle test driver disengage the autonomous mode and take immediate manual control of the vehicle. They used a simulator to study the eventual interactions between the surrounding entities and the AVs to evaluate crash risks. Out of the 69 manual disengagements reported by Google they found that 13 would have resulted in collisions and the rest of the 56 were safety critical, either due to the need for “proper perception of traffic lights, yielding appropriately to pedestrians and cyclists, and violations of traffic laws” (Google California DMV report). This provides further evidence for the role of trust in manual disengagements.

#### Identifying the sources of risk for disengagements

**Fig 3:**
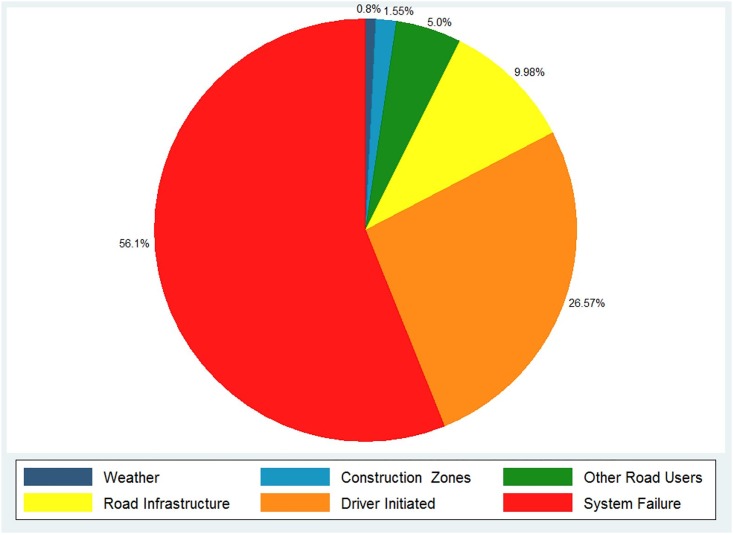
Reason for disengagement.

The companies also reported the cause for each of the disengagements. Fig 3 shows the reasons reported by all companies for disengagements from autonomous mode. The system failure was found to be the most common type of disengagement, which included hardware and software issues. This was followed by driver initiated disengagements. This should be expected based on the high correlation between the manual disengagement exposure and autonomous disengagement exposure.

Road infrastructure was also found to be a major cause for disengagements, and were attributed to improper detection of traffic lights, and poor road conditions such as improper lane marking, holes and bumps. Other road users such as cyclists, pedestrians, emergency vehicles and other drivers driving recklessly were also found to be a reason for disengagements. Finally, construction zones and weather due to rain and sun glare also caused disengagements.

### Accidents

The main argument for the adoption of automated vehicles has been predominantly hinged on improvement in safety. Table 1 lists the autonomous vehicle accidents that have occurred since the trials began (September 2014—February 2016). However, we only use data until November 2015 to study correlations with autonomous mile travelled, since the autonomous miles travelled was reported between September 2014 and November 2015.

**Table 1:** Summary sheet of accidents involving autonomous vehicles.

Date	Time	Company	AV mode?	Status of AV	Status of other vehicle	Type of collision	AV's fault?	Damage to AV	Damage to other vehicle	Injuries
14/10/2014	19:27	Delphi	Yes	Stopped	Moving	Side-swipe	No	damaged fender and front bumper	NA	No
26/02/2015	AM	Google	*	Moving	Moving	Side-swipe	No	right rear quarter panel and right rear wheel	NA	No
07/04/2015	AM	Google	Yes	Moving	Moving	Rear-end	No	minimal body damage	no damage	No
27/04/2015	16:27	Google	Yes	Stopped	Moving	Side-swipe	No	no damage	no damage	No
30/05/2015	12:00	Google	Yes	Stopped	Moving	Rear-end	No	minor damage to rear sensor and bumper	no damage	No
04/06/2015	08:54	Google	Yes	Stopped	Moving	Rear-end	No	no damage	no damage	No
18/06/2015	11:15	Google	Yes	Stopped	Moving	Rear end	No	scrapes to rear bumper	scrapes to front bumper	No
01/07/2015	17:16	Google	Yes	Stopped	Moving	Rear-end	No	minor damage to rear bumper	significant damage to front end	AV's driver and passenger reported whiplash, while the other driver reported neck and back pain
20/08/2015	09:36	Google	*	Moving	Moving	Rear-end	No	minor damage to rear bumper	moderate damage to front end and was towed	AV driver reported minor back pain
02/11/2015	14:30	Google	Yes	Stopped	Moving	Rear-end	No	minor damage to rear bumper	minor damage to headlight, vehicle hood, and front bumper	No
08/01/2016	13:41	Cruise Automation	*	Moving	Stopped	Side-swipe	No	Minor damage to front right quarter panel	Minor damage to front left quarter panel	No
14/02/2016	PM	Google	Yes	Moving	Moving	Side-swipe	Yes	Damage to left front fender	No damage	No

* Indicates the autonomous mode was manually disengaged few moments prior to the accident.

#### Descriptive analysis of autonomous vehicle accidents

All the accidents reported are shown in Table 1, and have occurred at low speeds in the vicinity of intersections on urban streets. Out of the twelve reported accidents, only one of them involved the autonomous vehicle being at fault. This was attributed to the autonomous vehicle incorrectly expecting the other vehicle to give way. In most of the cases where the other vehicle was determined to be at fault, the underlying cause could be attributed to the driver of the other vehicle expecting the autonomous vehicle to behave differently from what they would have normally expected. This points to the need to better understand driving interactions, and what are the behaviours that drivers expect from the vehicles they are interacting. Fortunately, most of the accidents were minor, and no serious injuries were reported. All collisions were either rear-end or side-swipe collisions.

#### Relationship between accidents and autonomous miles travelled

**Fig 4:**
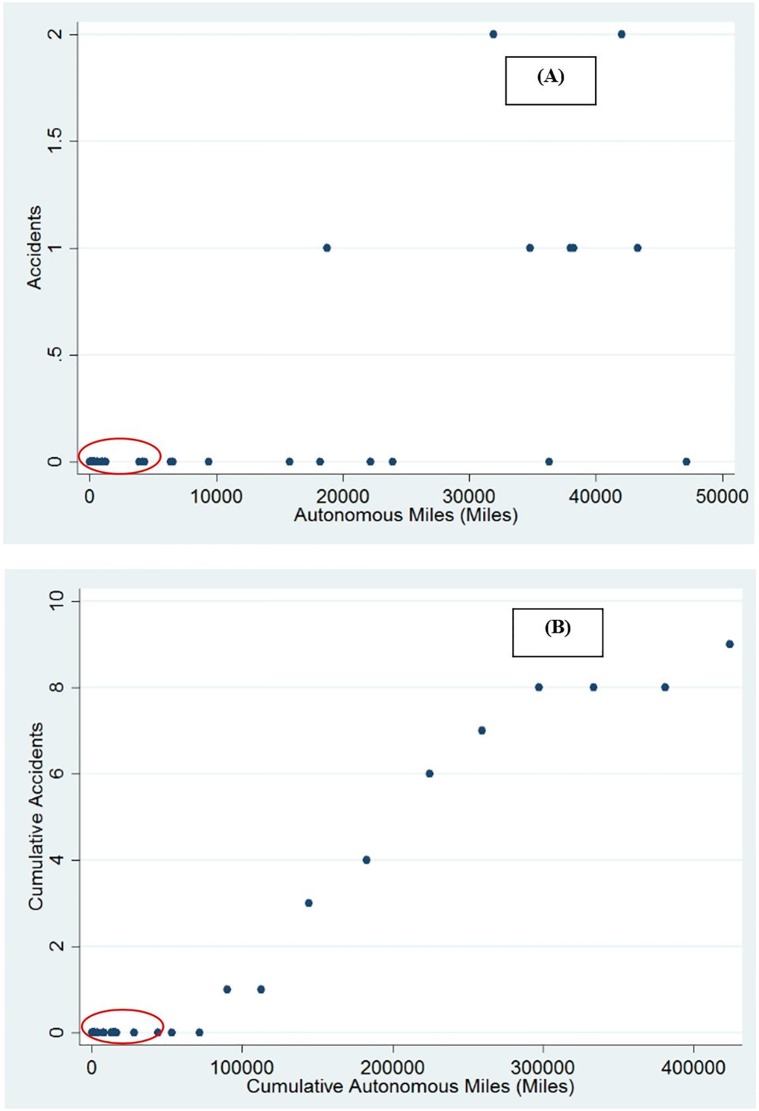
Relationship between accidents and autonomous miles. (A) Monthly Accidents vs. Autonomous miles. (B) Cumulative Accidents vs Autonomous miles.

As discussed earlier, accidents observed during autonomous driving should be expected to be related to autonomous miles travelled. To study this, we considered the monthly autonomous miles travelled by cars of each company and the accidents experienced by these companies. Fig 4A shows the relationship between monthly accidents and the autonomous miles travelled, and a strong positive correlation of 0.73 (p-value<0.01). Since nine out of the ten accidents during the reporting period were related to Google vehicles, a separate correlation analysis of monthly accident and vehicle miles only for Google cars also revealed a statistically significant correlation of 0.49 (p-value = 0.065). The correlation between the cumulative accidents and cumulative autonomous miles was even higher at 0.98 (p-value<0.01) for all vehicles, as well as only for google. The data points circled in red in Fig 4 represent the data points that do not belong to Google. This suggests that autonomous vehicle miles could be considered as an exposure for accidents associated with autonomous vehicles.

The autonomous miles travelled captures the exposure of the autonomous vehicle to be involved in an accident. These miles travelled captures the exposure to the random probability of being involved in a crash either due to other vehicles being at fault or the AV failing. To benchmark the crash exposure of AVs with normal vehicles, the California Highway Patrol (CHP) Safety Database from 2014 was used [11]. It was found that approximately 1 crash is expected every 2.07 million miles, however, based on data released by Google on their trials 1 crash was expected every 47,148 miles travelled by Google AVs. However, no fatalities occurred as compared to 1 death for every 108 million miles in California. Though the crash rate for Google AVs is higher than regular vehicles, there are a few caveats: 1) there is a high level of uncertainty in the crash rate estimates for Google AVs, due to the small sample size. 2) These crashes occurred as part of trials where vehicles were being tested to gather data. Clearly, there is a need for more data collection to improve the safety of AVs. 3) Despite being trials during the analysis period, there were no injury or fatal crashes. 4) The CHP only reports crashes on State, US, and interstate roads. However, AVs are required to report any crash on all types of roads.

### Reaction Times

At the time of the disengagement of the autonomous system, the driver of the vehicle needs to take control of the vehicle. The reaction time is measured based on the California DMV rule as the “period of time elapsed from when the autonomous vehicle test driver was alerted of the technology failure, and the driver assumed manual control of the vehicle”. Hence, this measurement does not include the perception time. The reaction times provide an understanding of how quickly an individual would react to a risk, and is a critical component of accident avoidance.

#### Reaction times for system failures by company

**Fig 5:**
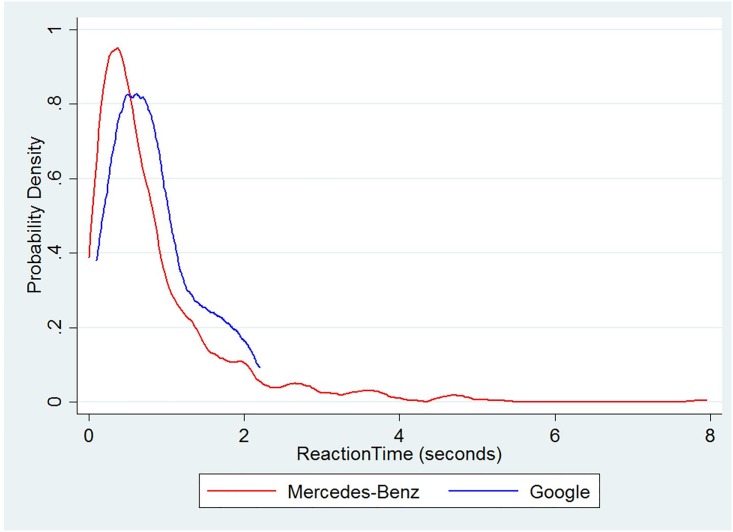
Probability density of reaction times for AVs of Mercedes-Benz and Google.

The probability distribution of the reaction times for system failures in Mercedes-Benz and Google is shown in Fig 5. The distribution of the reaction times is very similar, and no statistically significant difference was observed in the reaction times between the two companies. The distribution was estimated using an Epanechnikov kernel function with 50 bins [12]. The mean and standard deviation of the reaction times are shown in Table 2.

**Table 2:** Statistics of reaction time by company.

Company	Reason for disengagement	Number of observations	Reaction time	
			Mean	Std. Dev.
Google	System Failure	165	0.83	0.53
Mercedes Benz	System Failure	487	0.84	0.90

The mean of the reaction times was found to be approximately 0.83 seconds for both Mercedes-Benz and Google, with no significant statistical difference observed in the means (based on t-tests). Incidentally, Fambro et al. [13] also found the reaction time for braking in test vehicles to be 0.82 seconds. This is also consistent with other studies on reaction times [14]. Further, they found that the reaction time to brake increased by approximately 0.27 seconds if they owned the car. Considering that the autonomous vehicles were test vehicles, and none of the drivers actually held them, the reaction times to take over due to disengagement is remarkably close to the reaction time to brake. It should also be expected that the reaction times of individuals would increase as they begin to own autonomous vehicles.

#### Reaction times by cause and road type

**Fig 6:**
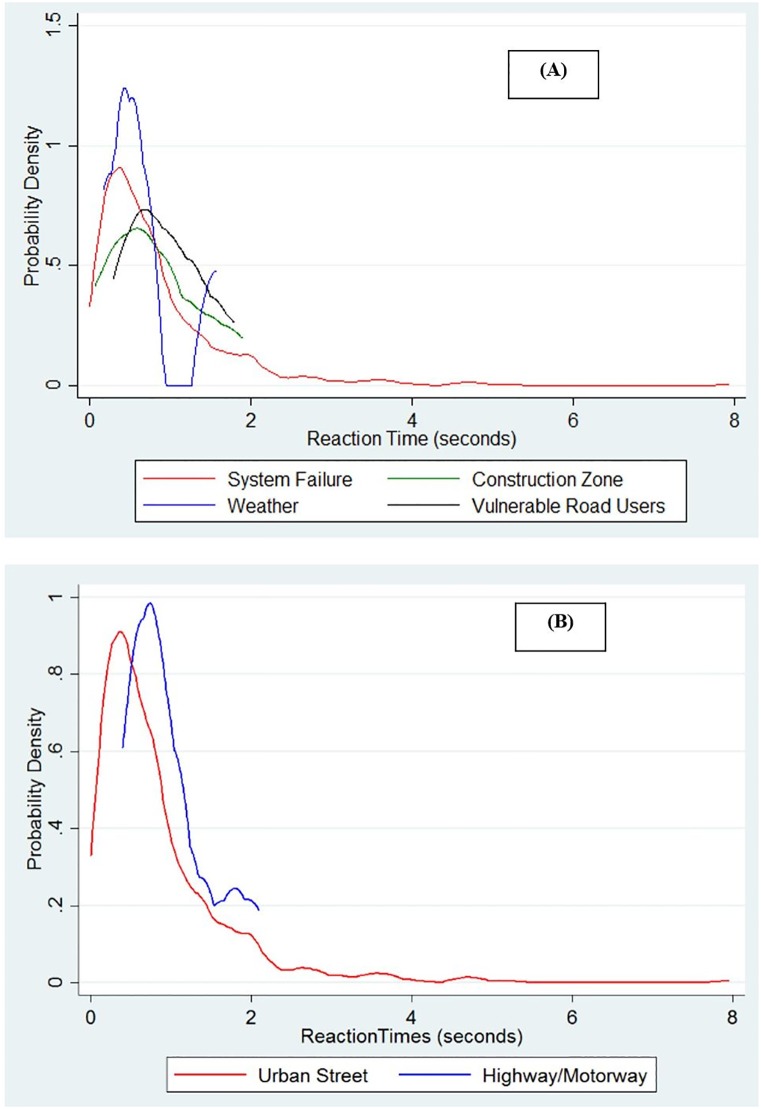
Probability density of reaction times. (A) Probability density by cause. (B) Probability density by type of roadway.

Given that the distribution of the reaction times for the two vehicle types were not statistically different for system failures, the reaction time data for the two companies were pooled to study the impact of various causes for failures and type of roadway on reaction times. The probability distribution of the reaction times at various failures is shown in Fig 6A. The statistics are presented in Table 3. No statistically significant differences (based on t-test) were observed in the mean of the reaction times between the different causes. This might be due to the small sample size in the reaction time data for disengagements caused due to construction zones, weather and road users (eg. cyclists, pedestrians and erratic behaviour of other cars).

**Table 3:** Statistics of reaction times based on causes and different road types.

Reason for disengagement	Number of observations	Reaction time	
		Mean	Std. Dev.
System failure	652	0.84	0.82
Adverse weather conditions	10	0.82	0.58
Road users	11	0.93	0.48
Construction zones	5	0.67	0.54
Streets (only system failure)	639	0.83	0.83
Highway/Motorway (only system failure)	13	1.00	0.50

The distribution of the reaction times for urban streets and non-urban streets (Highways and motorways) are shown in Fig 6B. Though the modes for the reaction time for the urban street was found to be approximately half of that on non-urban streets (mode of distribution of reaction time on urban streets is 0.37 and mode for non-urban streets is 0.79). However, though the means of the reaction time on highways/motorways was higher than urban streets (Table 3), no statistically significant differences were observed based on a t-test, which could also be attributed to the small number of observations on highways/motorways.

#### Correlation between reaction and autonomous vehicle miles travelled

As the vehicle miles travelled increases, the technology has improved, and hence the drivers trust in the system increases, which would eventually result in an increase in reaction time. To test this hypothesis, we study the statistical significance of the correlation between the reaction times and the cumulative monthly autonomous vehicle miles travelled.

Statistically significant positive correlations at a 95% confidence level were observed between the cumulative vehicle miles travelled by the company and the reaction times for Mercedes-Benz (0.122, p-value = 0.007) and Google (0.161, p-value = 0.029). This suggests that with increased miles driven the reaction times increase, which can be attributed to driver’s trust in the system. This increased reaction time and trust could be attributed to both improvements in technology as well as increased comfort level due to experience. The findings are consistent with the hypothesis.

## Conclusions

Fully automated cars will allow drivers to be driven by an informatics system in their own vehicle, which facilitates the drivers to engage in non-driving related activities. However, under unfortunate situations of system failure, the drivers are expected to react in an appropriate and timely manner to resume manual driving. It is essential to understand the causes for disengagements and the resulting driver reaction times as the AV technological development is at full pace. The data from the first year of reporting from the autonomous vehicle trials in California have provided insights into disengagement and accident exposure, perception-reaction time and trust.

Autonomous miles driven were found to have significantly high correlation and trends with accidents, suggesting that this quantity can be potentially used as a measure of exposure for disengagements and accidents. The reaction times to take control of the vehicle in the event of a disengagement was remarkably stable across the autonomous vehicles run by two different companies at 0.83 seconds. However, there were differences observed in reaction times based on the type of disengagements, type of roadway and autonomous miles travelled. This was also found to be consistent with the reaction times observed for braking in earlier studies. We also find evidence suggesting that the lack of trust can result in increased likelihood to manually take control of the vehicle and reduction in reaction times.

This study provides initial insights into the sources of risks for disengagement, correlation between accidents and autonomous miles travelled, which could help develop safety performance functions for autonomous driving, as well as the impact of different factors (road type, the cause of disengagements and experience) on reaction times. These new insights would inform practitioners about factors that need to be considered while planning for the advent of pervasive autonomous vehicles. Furthermore, the findings from this study offers several potential research extensions in engineering and psychology. For example, AVs are found to offer an increased level of trust and minimal cognitive load on the drivers. While this observation implies drivers gain confidence over AV system on one hand, the other school of thought would find this worrying on the grounds of safety issues & "driver-in-the-loop" concern. The findings also bring out the need to revisit roadway design manuals and safety manuals which still use the reaction time values that were determined empirically for manually operated vehicles. It is necessary to provide an adequate roadway infrastructure that makes AV operation safe and efficient at the entire network level. Thus, the questions discussed above need to be thoroughly addressed beforehand to prepare for a widespread introduction of AVs. In addition, the findings from this study will also lead to further innovation in vehicle automation and automobile engineering. Providing AV systems that can effectively interact with its environment can lead to a considerable reduction in the number of accidents.

Apart from further studying the phenomena presented in this paper, it is critical also to explore the impact of different types of human to machine interfaces to keep drivers engaged and alert. We also identify that further research is required to better understand the role of trip length, the interaction between drivers’ and their expectations from surrounding drivers, which was a primary cause for many of autonomous vehicle crashes.

## References

[1] Anderson JM, Kalra N, Stanley KD, Sorensen P, Samaras C, Oluwatola OA. Autonomous Vehicle Technology [Internet]. 2014 [cited 3 Nov 2015]. http://www.rand.org/pubs/research_reports/RR443-1.html

[2] Payre W, Cestac J, Delhomme P. Intention to use a fully automated car: Attitudes and a priori acceptability. Transp Res Part F Traffic Psychol Behav. 2014;27, Part B: 252–263.

[3] Rödel C, Stadler S, Meschtscherjakov A, Tscheligi M. Towards Autonomous Cars: The Effect of Autonomy Levels on Acceptance and User Experience. Proceedings of the 6th International Conference on Automotive User Interfaces and Interactive Vehicular Applications. New York, NY, USA: ACM; 2014. p. 11:1–11:8.

[4] Litman T. Autonomous Vehicle Implementation Predictions: Implications for Transport Planning. 2015. http://trid.trb.org/view.aspx?id=1338043

[5] Shladover S, Su D, Lu X-Y. Impacts of Cooperative Adaptive Cruise Control on Freeway Traffic Flow. Transp Res Rec J Transp Res Board. 2012;2324: 63–70.

[6] Fagnant DJ, Kockelman K. Preparing a nation for autonomous vehicles: opportunities, barriers and policy recommendations. Transp Res Part Policy Pract. 2015;77: 167–181.

[7] Helldin T, Falkman G, Riveiro M, Davidsson S. Presenting System Uncertainty in Automotive UIs for Supporting Trust Calibration in Autonomous Driving. Proceedings of the 5th International Conference on Automotive User Interfaces and Interactive Vehicular Applications. New York, NY, USA: ACM; 2013. pp. 210–217.

[8] Merat N, Jamson AH, Lai FCH, Daly M, Carsten OMJ. Transition to manual: Driver behaviour when resuming control from a highly automated vehicle. Transp Res Part F Traffic Psychol Behav. 2014;27, Part B: 274–282.

[9] Martens MH, van den Beukel AP. The road to automated driving: Dual mode and human factors considerations. 2013 16th International IEEE Conference on Intelligent Transportation Systems—(ITSC). 2013. pp. 2262–2267.

[10] Testing of Autonomous Vehicles [Internet]. [cited 8 Feb 2016]. https://www.dmv.ca.gov/portal/dmv/detail/vr/autonomous/testing

[11] CHP-SWITRS [Internet]. [cited 7 Jul 2016]. http://iswitrs.chp.ca.gov/Reports/jsp/RawData.jsp

[12] Silverman BW. Density Estimation for Statistics and Data Analysis. Boca Raton: Chapman and Hall; 1986.

[13] Fambro DB, Fitzpatrick K, Koppa RJ. Determination of Stopping Sight Distances. Transportation Research Board; 1997.

[14] Koppa RJ. Human Factors Revised Monograph on Traffic Flow Theory. National Research Council, Washington DC, USA; 2005.

